# Chapter 2 Knowledge Graphs: The Layered Perspective

**DOI:** 10.1007/978-3-030-53199-7_2

**Published:** 2020-06-20

**Authors:** Luigi Bellomarini, Emanuel Sallinger, Sahar Vahdati

**Affiliations:** 8 Institute Mihajlo Pupin, University of Belgrade, Belgrade, Serbia; 9 ADAPT SFI Centre, O’Reilly Institute, Trinity College Dublin, Dublin, Ireland; 10 CEPLAS, Botanical Institute, University of Cologne, Cologne, Germany; 11 Institute of Logic and Computation, Faculty of Informatics, TU Wien, Wien, Austria; 12 Banca d’Italia, Rome, Italy; 13 TU Wien, Vienna, Austria; 14 University of Oxford, Oxford, UK

## Abstract

Knowledge Graphs (KGs) are one of the key trends among the next wave of technologies. Many definitions exist of what a Knowledge Graph is, and in this chapter, we are going to take the position that precisely in the multitude of definitions lies one of the strengths of the area. We will choose a particular perspective, which we will call the layered perspective, and three views on Knowledge Graphs.

## Introduction

*Knowledge Graphs* (KGs) are one of the key trends among the next wave of technologies [340]. Despite the highlighted role in practice as well as research, and the variety of definitions of the notion, there is still no common understanding of what a Knowledge Graph *is*. In this introduction, we are *not* going to choose one definition of Knowledge Graphs. Many great introductions exist to particular definitions, and we will refer to some of them in this chapter. Instead, we are going to take the position that precisely in the *multitude of definitions* lies one of the *strengths* of the area.

At the same time, our aim is not towards a fully exhaustive, historical account of the evolution of Knowledge Graphs both regarding the term and the concept. Again, excellent historical and exhaustive accounts already exist, and we will refer to some of them in this chapter. Instead, we will choose a particular perspective, which we will call the *layered perspective*, and *three views* on Knowledge Graphs.

**Views on Knowledge Graphs.** While many ways of classifying types of Knowledge Graphs used in literature are possible, here we concentrate on the following three views:

- **knowledge representation tools:** where the focus is on how a Knowledge Graph is used to represent some form of knowledge.
- **knowledge management systems:** where the focus is the system managing the Knowledge Graph, similar to how database management systems play this role for databases.
- **knowledge application services:** where the focus is on providing a layer of applications on top of a Knowledge Graph.

**Fig. 1.**
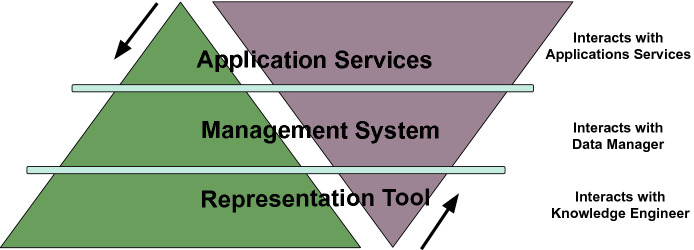
Ordered pyramids of views on KGs.

**The Layered Perspective.** While these three views certainly have independent value, they are most interesting when put *together as layers*: on the first layer is the **representation** of knowledge, on the middle layer is the **management** system for this knowledge, and on the top layer the **application** that it solves. This is illustrated in Fig. 1. There are three additional factors at play here:

- There are generally two ways of looking at the order of these layers. Some communities tend to see it **top-down** with the *application* that the KG solves as the focus, others tend to see it as **bottom-up**, with the *representation* of knowledge as the focus. Interestingly, there is even another one, as the data management community often sees the *management* system in the middle as the focus.
- The **borders** between these layers are fuzzy. Many academic and industrial systems cover two or three of these layers. In some cases, representation tools partly fulfill some of the characteristics of management systems. The same applies for application platforms.
- The central aspect of **reasoning** poses vastly different requirements to the three layers. Chapter 10.1007/978-3-030-53199-7_6 will be fully dedicated to this aspect.

Of course, it is clear that to achieve a great overall system, all layers and their interactions have to be taken into account; it is hardly possibly to provide a good knowledge application platform if the knowledge representation layer is not fit for the purpose.

**Organization.** The first three sections cover the three views we introduce above. In Sect. 2, we consider the view of KGs as knowledge representations tools; in Sect. 3, we consider the view of KGs a knowledge management systems; and in Sect. 4, we consider the view of KGs as knowledge application platforms. We will conclude with a section on challenges and opportunities.

## KGs as Knowledge Representation Tools

One of the most common views on Knowledge Graphs, which covers most of the given definitions, is to primarily view them as knowledge representation tools. In this section, we will give an overview of some of the notions with a particular focus on how they fit into the layered view.

Common to all these definitions is that, somewhat unsurprisingly given the term *Knowledge Graph*, there is *some* form of graph encoded by the formalism, and there is *some* form of knowledge encoded in it. Yet, in terms of **graphs**, what they widely differ is in whether a simple graph is the primary structure or whether we are actually dealing with richer settings where e.g., the graph has attributes associated to nodes or edges of the graph, or whether we are actually dealing with a hyper-graph (similar to full relational structures). Similarly, in terms of **knowledge**, what they widely differ is whether the graph *is* the knowledge, or the knowledge actually *generates* the entirety or parts of the graph. In some of the particular communities of computer science, Knowledge Graphs are explicitly considered as collections of facts about entities, typically derived from structured data sources such as Babelnet, OpenCyc, DBpedia, Yago, Wikidata, NELL and their shared features FreeBase [377]. In this way, a collection of facts represented in different languages but in the same structure is called a KG.

Critically though, forming a bridge to what we discussed in the introduction, in many cases these differences are only at the surface, and are often a question of representation, rather than fundamental. For example, it is clear that an arbitrary relational structure – or, in fact, an arbitrary data structure – can be encoded as a graph, and vice versa. Similarly, it is in many cases not a fundamental difference whether technically knowledge is encoded into the graph, into a separate knowledge representation language, or provided via other AI and ML frameworks. Still, fundamental differences do remain between different notions of Knowledge Graphs, and as we mentioned in the beginning, it is our position that these multifaceted definitions are one of the strengths of the field. In this section, we will explore such different definitions of Knowledge Graphs, highlighting both their commonalities and differences.

**Views on KGs as Representation Tools for Data.** The following definitions are pointing to the data structure in the representation. They mostly take a graph representation as a baseline and provide different explanations of how the graph structure helps with mapping real world information.

**A Mathematical Structure.** This is often considered to be the first recorded appearance [399] of the term “knowledge graph” – though not necessarily the concept of “knowledge graph”. Here, capturing knowledge from the real world as a teaching-leaning process is considered a way of building a graph of knowledge. In this work, prerequisites of learning are a necessary set of knowledge units that should usually be taught to the learner (human or machine) before. In this paper, a knowledge graph is essentially defined as:

A mathematical structure with vertices as knowledge units connected by edges that represent the prerequisite relation. – Marchi and Miquel, 1974 [298]

Although this definition has been given in the context of interactive learning between students and teachers, the concept can very well be adjusted for current machine learning and machine teaching [488] approaches where Knowledge Graphs are considered as the base of intelligence. In this definition, the degree of abstraction is hidden in the mathematical representation of knowledge in nodes as knowledge units and edges as connectors. Obviously, a specific language or data structure is not discussed due to its different context – so in our layer of *knowledge representation tools*, it is certainly a very abstract form of representation. It is roughly mentioned that knowledge units of a course for students to learn are represented as nodes of a graph in a game-theoretic way. And the links between the modes connect the knowledge units where the students can follow learning paths. In this way, the idea of representing common knowledge in a graph-based structure works in a similar way between this definition and today’s KGs. Similar to this view is also represented quite at the same time [387] where the teacher or the student can be replaced by a computer. It is argued that the directed graph in which the knowledge is represented in nodes and labeled links can influence the learning process for data analysis purposes.

**A Set of Justified True Beliefs.** In a tutorial by Microsoft, Yuqing Gao [146] follows Plato’s tripartite definition of knowledge as a subset of “Justified true beliefs” such that knowledge contains a truth condition, a belief condition and an inference of the former two that leads to justification of that. As example of such a “Justified true belief” is: *A is True*. *B knows A*. *B is justified in knowing A*. Knowledge in KGs is represented as triples of *(Subject, Predicate, Object)*, where Subject and Object are pointing to entities and Predicate represents the relation. A graph constructed from such triples contains nodes and edges where the nodes are pointing to entities as subject and object and the edges are for relations as predicates. There is extra information such as the metadata of each entity, which are shown as attributes. Following this, a set of key concepts for Knowledge Graphs as knowledge representation tools are introduced as:

- **Entity:** as real world entities
- **Edge:** relations of entities in a schema
- **Attribute:** metadata about an entity
- **Ontology:** definition of possible entities, relations and attributes

– Yuqing Gao, 2018 [146]

In this definition, two components of attribute and ontology are the concepts considered extra than other graph-based views. In fact, considering these components for knowledge representations adds on the characteristics of KGs. Entities and relations usually capture information stored in a Knowledge Base (KB).

**An Unambiguous Graph.** As seen before, most of the attempts in defining Knowledge Graphs have a focus on defining KGs as representing knowledge in a graph structure. Therefore, the KGs are often represented by the main components of a graph, namely nodes and edges. This graph is often considered as a directed and labeled graph, without which the structure of the graph cannot encode any significant meaning. When the nodes and edges are unambiguously unidentifiable, the graph is considered to be an unambiguous graph. With this foundation, a Knowledge Graph can be defined as:

“An Unambiguous Graph with a limited set of relations used to label the edges that encodes the provenance, especially justification and attribution, of the assertions.” – McCusker et al., 2018 [304]

This definition tried to go beyond representing KGs only as nodes and relations. In order to fulfills this definition, all the knowledge units of a KG including relations and nodes should be globally identifiable. In addition, the meaning of limited set of relations is followed from [440] meaning a core set of essential classes and relations that are true regardless of context. This level of abstraction is similar to data representation in triple format with unique resource identifiers.

**World Knowledge Graphs and Metadata.** At a basic level of observation, data represents elements as raw values collected from real-world domains of knowledge. Metadata represent information about the underlying data in a second abstract level. In order to represent knowledge from real world:

1. the real world objects need to be observed at least once and represented as data,
2. previous representation of such data is required to be captured as metadata and
3. all of these meta-level definitions on top of the abstractions of the objects of prime interest need to be connected.

At the formal and technical level, a formal and mathematical data structure, degree of abstraction, and a syntactic and semantic language are needed. Thus, characteristics of Knowledge Graphs lead the majority of the community to see and define them as tools for representing world knowledge in a graph model, where entities are represented as nodes and relations among entities are represented as directional edges. More formally, let $\mathcal {E} = \{ e_1, \cdots , e_{N_e} \}$ be the set of entities, $\mathcal {R} = \{r_1, \cdots , r_{N_r} \}$ be the set of relations connecting two entities, $\mathcal {D}= \{d_1, \cdots , d_{N_d} \}$ be the set of relations connecting an entity and a literal, i.e., the data relations, and $\mathcal {L}$ be the set of all literal values. Then:

“a knowledge graph $\mathcal {KG}$ is a subset of $(\mathcal {E} \times \mathcal {R} \times \mathcal {E}) \cup (\mathcal {E} \times \mathcal {D} \times \mathcal {L})$ representing the facts that are assumed to hold.” – Wang et al., 2014 [462].

However, there are different attempts in defining the concept of KGs that we will present in the following parts of this section.

**Views on KGs as a Representation Tool for Knowledge.** The following definitions are pointing to a view where the structure of the graph representation is not the only advantage but also includes ontological aspects of knowledge. The actual knowledge lies in the power of ontologies represented in the graph alongside the data level. In this way, the representation is enriched to handle the complexity of real world (not yet complete in coverage) and to empower learning, reasoning and inference abilities.

**A Particular Kind of Semantic Network.** The more intensive use of the term Knowledge Graphs starts from the early 1980s where the concept of Semantic Networks was introduced [13, 410, 482]. Later it was continued as a project by two universities from the Netherlands named Knowledge Graph [333, 449]. Following the definition of semantic networks as a specific structure of representing knowledge by labelled nodes and links between these nodes, KGs are defined as follows:

A knowledge graph is a kind of semantic network representing some scientific theory. – Popping, 2003 [357]

In this view, representation of explicit knowledge is considered by way of its formulation (logical or structured) [372]. While knowledge can be represented in multi modals such as text, image etc., this definition is applicable only on text extraction and analysis. Semantic networks are a way of structural formalism used for knowledge representation in nodes and edges. Such networks are mainly used in expert systems with a rule base language, a knowledge base sitting in the background, and an inference engine. Knowledge represented and reasoned by semantic networks are called author graphs with points as concept units representing meaning and labeled links between concepts. One essential difference between other views on Knowledge Graphs (in a broader sense) and the one derived from semantic networks is the explicit choice of only a few types of relations [219, 440].

**Representation of Human Knowledge.** Although many of the definitions for Knowledge Graph represent the concept as an formation representing tool, some views see KGs as a lingua franca of humans and machines. KGs contain information that is consumable by AI approaches in order to provide applications such as semantic search, question answering, entity resolution, and representation learning.

“A graph-theoretic representation of human knowledge such that it can be ingested with semantics by a machine; a set of triples, with each triple intuitively representing an assertion.” – Kejriwal, 2019 [237]

**Knowledge Represented with a Multi-relational Graph.** A large volume of human knowledge can be represented with a multi-relational graph. Binary relationships encode facts that can be represented in the form of RDF-type triples (head; predicate; tail), where head and tail are entities and predicate is the relation type. The combination of all triples forms a multi-relational graph, where nodes represent entities and directed edges represent relationships. The resulting multi-relational graph is often referred to as a Knowledge Graph. Knowledge Graphs (KGs) provide ways to efficiently organize, manage and retrieve this type of information, and are increasingly used as an external source of knowledge for problems like recommender systems, language modeling [2], question answering or image classification.

One critical point to emphasize is that while many of the KGs we see today contain as their *knowledge* mostly simple ground *data*, more and more applications need an *actionable knowledge* representation. To a certain extent, this is already the case of existing Knowledge Base Management Systems, backed by ontologies for which reasoning tasks are of different computational complexity and expressive power. The importance of supporting implicit knowledge becomes central for KGs as well, especially when they are a component of an Enterprise AI applications, to the point that intensional knowledge should be considered part of the KG itself. Consequently, reasoning, i.e., turning intensional into derived ground knowledge, becomes inherently part of the KG definition.

For example, in a financial Enterprise AI application, the body of regulatory knowledge and the functioning rules of the specific financial domain are of the essence. As another example, in a logistics setting, the knowledge of how particular steps in a supply chain interact is often more important than the pure data underlying the supply chain. Many more such examples could be given.

In total, it is clear that in modern KG-based systems a rich knowledge representation must be considered and properly handled in order to balance the increased complexity with many other relevant properties including usability, scalability, performance, and soundness of the KG application. We conclude with a relatively structured, concrete definition accounting for these aspects:

“A semi-structured datamodel characterized by three components: (i) a ground extensional component, that is, a set of relational constructs for schema and data (which can be effectively modeled as graphs or generalizations thereof); (ii) an intensional component, that is, a set of inference rules over the constructs of the ground extensional component; (iii) a derived extensional component that can be produced as the result of the application of the inference rules over the ground extensional component (with the so-called “reasoning” process).” – Bellomarini et al., 2019 – [40].

Here we focus on the knowledge representation aspects covered in this view and in further layers we will discuss how this definition also sees KGs as management systems and application platforms.

## KGs as Knowledge Management Systems

In this section, we present the view of Knowledge Graphs as knowledge management systems. The clear analogy to see here is what a database management system is for databases: A system to create, manipulate and retrieve data. What this adds to the previous section’s view of KGs as knowledge representation tools is the *service* that a KG as a knowledge management system has to offer. In particular, it has to provide support for the user to (i) add knowledge to a KG (ii) derive new knowledge using existing knowledge, and (iii) retrieve data through a form of general-purpose query language. In both (ii) and (iii), the aspect of *reasoning* with and about knowledge becomes essential, which we will discuss in detail in Chap. 10.1007/978-3-030-53199-7_6.

**A Network of All Kinds of Things.** One of the early attempts after the appearance KGs in 2012, was a work clarifying the meaning of taxonomy, thesaurus, ontology and Knowledge Graph [54]. These concepts have been used by scholars mostly without specific borderlines. In some cases, they even utilized interchangeably. Starting from the Simple Knowledge Organization System (SKOS) as a standard for building an abstract model, taxonomies are introduced as controlled vocabularies to classify concepts and thesauri to express associations and relations between concepts and their labels including synonyms. Ontologies are considered as complex and more detailed versions of those domain conceptualizations when the dependencies between concepts and relations get more specific. There are also rules and constraints defined for representing knowledge which refer to ontologies as explicit and systematic specification of conceptualization for any kind of existence. By this, in building an abstract model of the world or a domain, the meaning of all concepts must be formally defined that can be interpreted correctly by machines. There must also be consensus about the definition of the concepts such as the meaning in transferred correctly. In AI-based approaches, the existence of things is defined when they can be represented [172]. Following these concepts, finally Knowledge Graphs are introduced as enriched models around the aforementioned concepts more precisely:

“Knowledge Graphs could be envisaged as a network of all kinds of things which are relevant to a specific domain or to an organization. They are not limited to abstract concepts and relations but can also contain instances of things like documents and datasets.” – Blumauer, 2014 [54].

The motivation behind having KGs is expressed in posing complex queries over a broader set of integrated information from different source for knowledge discovery, and in-depth analyses. Knowledge Graphs being the networks of all kinds of information, the industry-scale of such integration, together with the inclusion of Taxonomy, Thesaurus and Ontology is seen as Enterprise Knowledge Graphs (EKGs). Since this definition is mostly using semantic web technologies, the specific querying language that suits this definition is suggested to be SPARQL, and Resource Description Framework (RDF) is used as the data and ontology representation model.

**A Graph-based Representation of Knowledge.** In a similar way, Knowledge Graphs are considered to be any kind of graph-based representations of general information from the world [348]. This includes consideration of other graph-based data models such as the RDF standard pushed by Semantic Web or any knowledge representation languages such as description logic (DL). A simple triple of such a graph representation could be seen as two nodes representing entities which are connected by a relation. There are also predefined structural relations such as *is a* relation which denotes the type of entities, or relations denoting class hierarchies. As discussed before, such relations are usually represented as ontologies. In a universally unified level, this allows interlinking of different datasets, which leads to big data in graph representations, or so called Knowledge Graphs. Overall, this view mostly follows the basics of semantic representation of knowledge bases on the Web. The community has never come up with a formal definition but generally, on a technical level, the overlapping concepts have been coined together and built up a general understanding of the concept connections. Following this view, a structured list of four characteristics has been listed such that “a Knowledge Graph:

1. mainly describes real world entities and their interrelations, organized in a graph,
2. defines possible classes and relations of entities in a schema,
3. allows for potentially interrelating arbitrary entities with each other,
4. covers various topical domains.” – Pullheim, 2017 [348]

Basically, the first characteristic refers to the terminological knowledge about concepts of a domain, and is represented as *TBox* in description logic. The second characteristic points to the assertions knowledge about individual entities as *ABox*. By such a definition, a DL knowledge base can be constructed, on top of which inference of new knowledge from the existence knowledge can be applied. More in common language, the ontologies without instances and the datasets without ontologies are not considered as a KG. As this way of knowledge representation involves logical rules and ontologies, the KG created by this has reasoning abilities. Complex queries are made possible with the power of data representation and the existence of ontologies. Thus, this definition also falls into the category of a KG being a management system.

**A Finite Set of Ground Atoms.** Looking at KGs as a graph of nodes and links, assuming $\mathcal {R}$ as a set of relations and $\mathcal {C}$ a set of entities, the following formal definition is given:

“A Knowledge Graph $\mathcal {G}$ is a finite set of ground atoms of the form *p*(*s*, *o*) and *c*(*s*) over $\mathcal {R} \cup \mathcal {C}$. With $\Sigma _g = \langle \mathcal {R}, \mathcal {C} \rangle$, the signature of *g*, we denote elements of $\mathcal {R} \cup \mathcal {C}$ that occur in *g*.” – Stepanova, 2018 [413]

This adopts first-order logic (FOL), seeing a set of correct facts as a KG. These facts are represented as unary and binary triples. In addition to the reasoning and querying power that comes from this definition, the power of explainability is also addressed here. Such features are a must now for KGs as management systems for AI-based downstream tasks.

**A Graph of Data with the Intent to Compose Knowledge.** In one of the attempts in (re)defining Knowledge Graphs [55], datasets are seen in graph representations with nodes representing entities and links denoting their relations. Example graph representation can be considered as:

- directed edge-labelled graphs as labelled edges between entities as nodes,
- property graphs as additional annotations on the edges,
- name graph as a collection of data represented in directed edge-labelled.

In a succinct view, the definition of KGs is then summarized as:

“A graph of data with the intent to compose knowledge.” – Hogan et al., 2019 [55]

This definition brings another management action into the picture, namely *composing knowledge*. This is not only about knowledge representation in a graph structure but also using that graph for a dedicated purpose. Construction of a KG under this definition means facilitating complex management steps.

**An Open-World Probabilistic Database** [58]**.** Probabilistic databases, often abbreviated PDBs, as the state of the art of processing large volumes of uncertain data in a complete platform which is a combination of methods from information extraction, natural language processing to relational learning [212].

“Knowledge Graphs are addressed as open-world Probabilistic databases (OpenPDBs).” – Borgwardt, 2017 – [58].

**A Knowledge Graph Management System** [42]**.** The authors pose a number of requirements or desiderata for a *Knowledge Graph Management System* (KGMS) in terms of the main system capabilities:

- *simple modular syntax:* easy to add and remove facts and rules
- *high expressive power:* at least as expressive as Datalog (i.e., full recursion)
- *numeric computation and aggregation:* real-world required features
- *ontological reasoning:* at least as expressive as SPARQL and OWL 2 QL
- *probabilistic reasoning:* should support a form of probabilistic reasoning
- *low complexity:* the core language should be tractable in data complexity
- *rule repository, management and ontology editor:* management facilities
- *dynamic orchestration:* allow orchestration of complex, real-world workflows

They also formulate a number of access/integration requirements, some of which are what we consider core capabilities in this section, some of which we will include in the following section on *application services*. The ones of core relevance for management systems are:

- *big data access:* must be able to consume Big Data sources and interface with such systems
- *database and data warehouse access:* must seamlessly integrate with relational databases, graph stores, RDF stores, etc.
- *ontology-based data access (OBDA):* allow queries on top of ontologies
- *multi-query support:* allow multiple queries executed in parallel to benefit from each other
- *procedural code support:* allow easy integration of procedural code

They subsequently presented the Vadalog system [38] in more technical detail, focusing on algorithms and data structures to meet the requirement on high expressive power, ontological reasoning and low complexity at the same time. Subsequent papers discuss highly parallelizable fragments [44, 45, 49], how to achieve maintainability [64] and other related topics, including more fundamental aspects [43, 162].

## KGs as Knowledge Application Services

While not usually providing quotable definitions of Knowledge Graphs, there is a huge body of work that does not primarily treat KGs as representation tools or management systems, but as a platform to provide a large number of crucial applications. So instead of a KG being used to *represent* information or *manage* information, it is rather the capability of the KG to natively or easily support certain applications that define what a KG is.

For example, [116] introduces KGs not only as the graph containing all of the Amazon product data, but as a graph that has the special capability of natively supporting entity resolution (i.e., knowing when two products are the same) and entity linking (i.e., knowing when two products or other entities are related). Similar considerations can be found in many KG-related fields. It could even be argued that the amount of work in KG completion, etc., makes this application-oriented view of KG the most important one.

Clearly, the border between the two views of management and application is debatable, and we *invite* the reader to critically think of what one *should* consider as an essential general-purpose service of a knowledge management system, and what should be part of an application service. We shall explore this aspect in this section, and in particular in Chap. 10.1007/978-3-030-53199-7_6. For example, while *question answering* in our opinion would typically be considered as an application service, as would be offering *recommender system* capabilities, it is less clear for relatively general-purpose application services such as *entity resolution* and *link prediction*, which could be seen as a requirement of a *general purporse* knowledge management system. Here, we will consider all of four of these as application services as they clearly offer a *well-defined* application compared to a management system offering a query language that supports such applications.

**Knowledge Organization System.** This view is from the domain of libraries and humanities where KGs are sees as knowledge organization systems. Even in a further vision, KGs are seen to integrate the insights derived from analysis in large-scale domains. This vision is already in practice by reasoning systems considered as a part of the KG concept.

“Knowledge Graphs represent concepts (e.g., people, places, events) and their semantic relationships. As a data structure, they underpin a digital information system, support users in resource discovery and retrieval, and are useful for navigation and visualization purposes.” – Haslhofer, 2018 [188]

Scholarly communication artifacts, such as bibliographic metadata about scientific publications, research datasets, citations, description of projects,and profile information of researchers, has recently gained a lot of attention with KG technologies. With the help of Linked Data technologies, interlinking of semantically represented metadata has been made possible. Discovering and providing links between the metadata of scholarly artifacts is important in scholarly communities. This definition has a particular view of KGs for such purposes. The links are generated retrospectively by devising similarity metrics over sets of attributes of the artifact descriptions. Interlinking of such metadata provides shareable, extensible, and easily re-usable metadata in the form of KGs. We also address the scholarly domain as one of the example applications.

**Rule Mining and Reasoners.** One of the early attempts in systematic definitions of KGs goes beyond seeing them as only a representation tool but more as a management system close to database management systems.

“A Knowledge Graph acquires and integrates information into an ontology and applies a reasoner to derive new knowledge.” – Ehrlinger, 2016 – [121].

This is one of the early attempts in defining KGs in a systematic way with a different view. Similarly, the following definitions sees KGs as a specific data model. There are several rule mining reasoners around which are purely designed to consume the ontology and mine relational patterns out of the KG. One example of this category is AMIE [144]. We categorize it under this view because it is more than just a representation tool and performs some data management steps. It has RDF as the data model for representing the facts and rules and uses its own internal functions for rule mining.

**Data Application Platform.** The VADA project [257] saw many application services built on top of its Knowledge Graph Management System (KGMS) Vadalog [164]. Before going into concrete examples, let us inspect the application service requirements given in [42]:

- *data cleaning, exchange, integration:* often summarized as “data wrangling”
- *web data extraction, interaction and IoT:* to interact with the outside world
- *machine learning, text mining, NLP, data analytics:* providing and interfacing with external such services. An interesting side-note is that the authors here invert the perspective: it is not always the knowledge graph system providing the application service, but sometimes also using it.
- *data visualization:* for providing data consumable by an end-user or analyst

Let us now proceed to concrete examples of these abstract requirements. Prime among them is:

- *Data Wrangling*, i.e., the whole process of bringing raw data into an integrated format amenable to Big Data Analytics [141, 257, 258]. Further services seen as key were at the data acquisition phase the application service
- *Data Extraction* [132, 262, 308]. Further key application services are those of
- *Recommender Systems* [82], including services for downstream machine-learning applications which need feature engineering. A connected but independent application platform requirement is that of
- *Social Choice* [89, 90] where the application requirement is to choose among a number of different users’ preferences the best joint solution. A further one, for which it is somewhat debatable whether it is a management system requirement or an application service is that of
- *Machine Learning* [41] service integration - bridging typical KGMS services and machine learning services. Another interesting case is that of a vertical application service collection, namely that of
- *Company Knowledge Graphs* [24, 39], especially important for the COVID-19 perspective raised in one of the works on the economic impact of the pandemic.

## KGs in Practice: Challenges and Opportunities

The initial release of KGs was started on an industry scale by Google and further continued with the publication of other large-scale KGs such as Facebook, Microsoft, Amazon, DBpedia, Wikidata and many more. As an influence of the increasing hype in KG and advanced AI-based services, every individual company or organization is adapting to KG. The KG technology has immediately reached industry, and big companies have started to build their own graphs such as the industrial Knowledge Graph at Siemens [206]. In a joint work [331] for sharing ideas from large-scale industrial Knowledge Graphs, namely Microsoft, Google, Facebook, eBay and IMB, authors stated a broad range of challenges ahead of research and industry involving KGs. Despite the content-wise difference and similarities of those Knowledge Graphs, the discussions involve data acquisition and provenance problems due to source heterogeneity and scalability of the underlying managements system. Here we introduce the Enterprise Knowledge Graph of Italian companies for the Central Bank of Italy.

### Integrated Ownership and Company Control

The database at our disposal contains data from 2005 to 2018, regarding unlisted companies and their shareholders (companies or persons). If we see the database as a graph, where companies and persons are nodes and shareholding is represented by edges, on average, for each year the graph has 4.059M nodes and 3.960M edges. There are 4.058M Strongly Connected Components (SCC), composed on average of one node, and more than 600K Weakly Connected Components (WCC), composed on average of 6 nodes, resulting in an high level of fragmentation. Interestingly, the largest SCC has only 15 nodes, while the largest WCC has more than one million nodes. The average in- and out-degree of each node is ${\approx }{1}$ and the average clustering coefficient is ${\approx }{0.0084}$, which is very low compared to the number of nodes and edges. Furthermore, it is interesting to observe that the maximum in-degree of a node is more than 5K and the maximum out-degree is more than 28K nodes. We also observe a high number of self-loops, almost 3K, i.e. companies that directly own shares of themselves in order to subtract them from the market. The resulting graph shows a scale-free network structure, as most real-world networks [148]: the degree distribution follows a power-law and there are several nodes in the network that act as hubs.

The Register of Intermediaries and Affiliates (RIAD), the ownership network of European financial companies run by the European Central Bank, is a good example of the company control topology at the European level. It has one large SCC containing 88 nodes, and all the others with less than 10 nodes; there is one huge WCC, with 57% of the nodes, with the others scattered around small WCCs with 11.968 nodes on average and (apart from the largest one), none with more than 472 nodes.

### Large-Scale Scholarly Knowledge Graphs

The complexity of scholarly data fully follows the *6 Vs* of Big Data characteristics towards building Scholarly Knowledge Graphs [405]. The term Big Scholarly Data (BSD) [474] is coined to represent the vast amount of information about scholarly networks including stakeholders and artifacts such as authors, organizers, papers, citations, figures. The heterogeneity and complexity of data and their associated metadata distributed on the Web perfectly qualifies this domain for Big Data challenges towards building Scholarly KGs:

- Volume refers to the ability to ingest and store very large datasets; in the context of scholarly metadata, at least over 114 million scholarly documents [240] were recorded in 2014 as being available in PDF format. In computer science, the total number of publications of the different types is reaching 4 million [423]. Different types of publication in different formats are being published every day in other scientific disciplines.
- Velocity denotes the growth rate generating such data; the average growth rate of scientific publishing is measured as 8 to 9% [61].
- Variety indicates multiple data formats and models; the domain of scholarly communication is a complex domain [29] including many different types of entities with complex interrelationships among them.
- Value concerns the impact of high quality analytics over data; certain facts play enormously important roles in the reputation and basic life of research stakeholders. Providing precise and comprehensive statistics supports researchers with already existing success measurement tools such as number of citations. In additions, deep and mined knowledge with flexible analytics can provide new insights about artifacts and people involved in the scholarly communication domain.
- Veracity refers to the biases, ambiguities, and noise in data; this characteristic is especially applicable in the context of the scholarly communication domain due to deduplication problems [296] and the ambiguity problem for various scholarly artifacts as well as person names.
- Variability of the meaning of the metadata [474].

Discovering high quality and relevant research-related information has a certain influence on the life of researchers and other stakeholders of the communication system [109]. For examples, scholars search for quality in the meaning of fitness for use in questions such as “the venues should a researcher participate” or “the papers should be cited”. There are already attempts to assist researchers in this task, however, resulting in recommendations often being rather superficial and the underlying process neglecting the different aspects that are important for authors [439]. Providing recommendation services to researchers and a comprehensive list of criteria while they are searching for relevant information. Furthermore, having access to the networks of a paper’s authors and their organizations, and taking into account the events in which people participate, enables new indicators for measuring the quality and relevance of research that are not just based on counting citations [438]. Thus each of the Vs of Big Data needs careful management to provide such services for scholarly communities.

## Conclusion

In this chapter, we introduced Knowledge Graphs in a layered perspective: Knowledge Graphs as (1) knowledge representations tools, (2) knowledge management systems, and (3) knowledge application services. We did not focus on a single definition here but presented a multitude of definitions, putting them into the context of this layered perspective. We deliberately stopped short of the chapter being an exhaustive historical overview as excellent overviews have already been written.

We also pointed toward aspects of particular concern: The different ways that particular communities see KGs (top-down or bottom-up, or even middle-layer in focus). We concluded with the practical challenges of KGs by providing typical industrial and academic applications. Throughout the chapter, we discussed the aspect of *reasoning* being a natural counterpart to this “bigger picture” focus section, and we shall consider reasoning in greater detail in Chap. 10.1007/978-3-030-53199-7_6.

